# Multi-task bioassay pre-training for protein-ligand binding affinity prediction

**DOI:** 10.1093/bib/bbad451

**Published:** 2023-12-11

**Authors:** Jiaxian Yan, Zhaofeng Ye, Ziyi Yang, Chengqiang Lu, Shengyu Zhang, Qi Liu, Jiezhong Qiu

**Affiliations:** Anhui Province Key Lab of Big Data Analysis and Application, University of Science and Technology of China, JinZhai Road, 230026, Anhui, China; Tencent Quantum Laboratory, Tencent, Shennan Road, 518057, Guangdong, China; Tencent Quantum Laboratory, Tencent, Shennan Road, 518057, Guangdong, China; Anhui Province Key Lab of Big Data Analysis and Application, University of Science and Technology of China, JinZhai Road, 230026, Anhui, China; Tencent Quantum Laboratory, Tencent, Shennan Road, 518057, Guangdong, China; Anhui Province Key Lab of Big Data Analysis and Application, University of Science and Technology of China, JinZhai Road, 230026, Anhui, China; Tencent Quantum Laboratory, Tencent, Shennan Road, 518057, Guangdong, China

**Keywords:** bioassay, protein–ligand binding affinity, graph neural network, pre-training

## Abstract

Protein–ligand binding affinity (PLBA) prediction is the fundamental task in drug discovery. Recently, various deep learning-based models predict binding affinity by incorporating the three-dimensional (3D) structure of protein–ligand complexes as input and achieving astounding progress. However, due to the scarcity of high-quality training data, the generalization ability of current models is still limited. Although there is a vast amount of affinity data available in large-scale databases such as ChEMBL, issues such as inconsistent affinity measurement labels (i.e. IC50, Ki, Kd), different experimental conditions, and the lack of available 3D binding structures complicate the development of high-precision affinity prediction models using these data. To address these issues, we (i) propose Multi-task Bioassay Pre-training (MBP), a pre-training framework for structure-based PLBA prediction; (ii) construct a pre-training dataset called ChEMBL-Dock with more than 300k experimentally measured affinity labels and about 2.8M docked 3D structures. By introducing multi-task pre-training to treat the prediction of different affinity labels as different tasks and classifying relative rankings between samples from the same bioassay, MBP learns robust and transferrable structural knowledge from our new ChEMBL-Dock dataset with varied and noisy labels. Experiments substantiate the capability of MBP on the structure-based PLBA prediction task. To the best of our knowledge, MBP is the first affinity pre-training model and shows great potential for future development. MBP web-server is now available for free at: https://huggingface.co/spaces/jiaxianustc/mbp.

## INTRODUCTION

Protein–ligand binding affinity (PLBA) is a measurement of the strength of the interaction between a target protein and a ligand drug [1]. Accurate and efficient PLBA prediction is the central task for the discovery and design of effective drug molecules *in silico* [2]. Traditional computer-aided drug discovery tools use scoring functions (SFs) to estimate PLBA roughly [3], which is of low accuracy. Molecular dynamics simulation methods can achieve more accurate binding energy estimation [4], but these methods are typically expensive in terms of computational resources and time. Recent years have witnessed the successful application of deep learning (DL) in various bioinformatics tasks, such as protein structure prediction [5], anticancer peptides prediction [6] and lung cancer decision-making [7]. Considered as a promising tool for accurately and rapidly predicting PLBA, a series of DL-based scoring functions have been built, such as Pafnucy [8], OnionNet [9], Transformer-CPI [10], IGN [11] and SIGN [12]. In particular, structure-based DL models that use the 3D structure of protein–ligand complexes as inputs are most successful, which typically use 3D convolutional neural networks (3D-CNNs) [13–15] or graph neural networks (GNNs) [11, 12] to model and extract the interactions within the protein–ligand complex structures. However, the generalizability of these data-driven DL models is limited because the number of high-quality samples in PDBbind used for model training is relatively small ($\sim $5000) [16].

**Figure 1:**
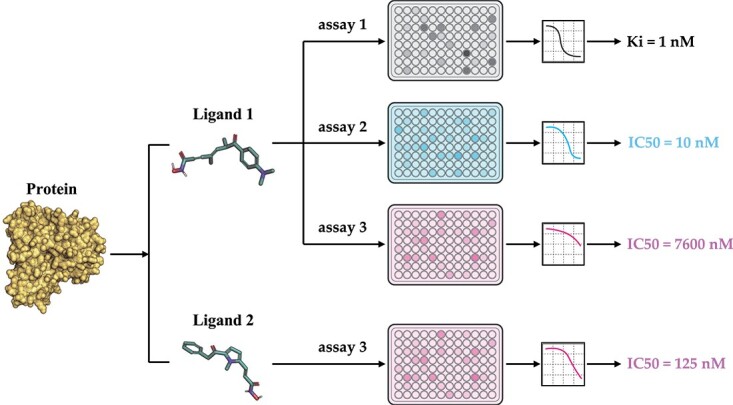
A real example of bioassay data in ChEMBL. (1) The top three panels show an example where the same protein–ligand pair have different binding affinities with assay 1–3 in terms of measurement type (IC50 versus Ki) and value (IC50=10 nM versus IC50=7600 nM). (2) The bottom two panels show an example of the binding of different ligands (Ligand 1 and 2) to a protein in the same assay (assay 3).

One solution for this problem is pre-training, which has been widely used in computational biologies, such as molecular pre-training for compound property prediction [17–20] and protein pre-training for protein folding [21–24]. These pre-training models utilize data from large-scale datasets to learn embeddings, which expand the ligand chemical space and protein diversities. Therefore, affinity pre-training models on a large amount of affinity data in databases such as ChEMBL [25] and BindingDB [26] can be helpful. Nevertheless, though attempts have been made to directly use the data, such as BatchDTA [27], several challenges have prevented researchers from widely using ChEMBL data for PLBA previously. Firstly, the data were collected from various bioassays, which introduce different system biases and noises to the data and make it difficult for comparison [27, 28] (’label noise problem’). For some cases, the affinities of the same protein–ligand pair in different bioassay can have a difference of several orders of magnitude (Figure 1). Secondly, several types of affinity measurement exist (’label variety problem’), such as half-maximal inhibitory concentration (IC50), inhibition constant (Ki), dissociation constant (Kd), half-maximal effective concentration (EC50), etc., which cannot be compared directly as well. Thirdly, the unavailability of 3D structures of protein–ligand complexes within ChEMBL poses a significant limitation for researchers in training and leveraging structure-based DL models (’missing conformation problem’).

To solve the above problems, we propose the Multi-task Bioassay Pre-training (MBP) framework for structure-based PLBA prediction models. In general, by introducing multi-task and pairwise ranking within bioassay samples, MBP can make use of the noisy data in databases such as ChEMBL. Specifically, the multi-task learning strategy [29] treats the prediction of different label measurement types (IC50/Ki/Kd) as different tasks, thus enabling information extraction from related but different affinity measurements. Meanwhile, although different assays can introduce different types of noises to the data, data from the same assay are relatively more comparable. Inspired by recent progress in the recommendation system [30–32], by considering ranking between samples from the same assay, the model is enforced to learn the relative relationship of samples and differences in protein–ligand interactions, which allows the MBP to learn robust and transferrable structural knowledge beyond the noisy labels.

We then construct a pre-training dataset, ChEMBL-Dock, for MBP. ChEMBL-Dock contains 313 224 protein–ligand pairs from 21 686 assays and the corresponding experimental PLBA labels (IC50/Ki/Kd). Molecular docking softwares are employed to generate about 2.8M docked 3D complex structures in ChEMBL-Dock. Then, we implant MBP with simple and commonly used GNN models, such as GCN [33], GIN [34], GAT [35], EGNN [36] and AttentiveFP [37]. Experiments on the PDBbind core set and the CSAR-HiQ dataset have shown that even simple models can be improved and achieve comparable or better performances than the state-of-the-art (SOTA) models with MBP. Through ablation studies, we further validate the importance of multi-task strategy and bioassay-specific ranking in MBP.

Overall, the contributions of this paper can be summarized as follows:

- We propose the first PLBA pre-training framework MBP, which can effectively utilize large-scale noisy data and significantly improve the accuracy and generalizability of PLBA prediction models.
- We construct a high-quality pre-training dataset, ChEMBL-Dock, based on ChEMBL, which significantly enlarges existing PLBA datasets in terms of chemical diversities.
- We show that even vanilla GNNs can significantly outperform the previous SOTA method by following the pre-training protocol in MBP.

## RELATED WORK

**PLBA prediction.** One critical step in drug discovery is scoring and ranking the predicted PLBA. Scoring functions can be roughly divided into four main types: force-field-based, empirical-based, machine-learning-based and DL-based [12].

Force-field-based methods aim at estimating the free energy of the binding by using the first principles of statistical mechanics [9]. Despite its remarkable performance as a gold standard, it suffers from high computational overhead.

Empirical-based methods [38–40] generally attempt to estimate the binding affinity by considering individual contributions that are believed to be significant, such as hydrophobic contacts, hydrophilic contacts or the number of hydrogen bonds [41]. These contributions are typically combined using a linear sum approach [42]. The empirical methods possess excellent explainability and have demonstrated numerous successful applications [40, 43]. However, the design of such methods requires expertise in the domain, and their performance has been limited due to oversimplifications of certain physical interactions [41, 44].

Machine-learning-based methods aim to predict binding affinity based on a data-driven learning paradigm. Machine learning techniques, such as random forest [45] and support vector machines (SVMs) [46], are exploited to extract useful information from big databases [47]. These methods, such as RF-Score [45], already show superior performance on prediction accuracy compared with classical methods, while the reliance on predefined rules and/or descriptors might introduce bias of domain expertise and prevent an end-to-end learning manner from raw inputs [11].

Recently, due to advances in DL methods and the creation of structure-based protein–ligand complex datasets, many structure-based DL methods [8, 9, 11, 12, 18, 48–51] have been developed for predicting binding affinity. Such methods directly learn the structural information of protein–ligand complexes end-to-end, avoiding artificial feature design. However, due to the scarcity of high-quality training data, current methods still suffer from poor generalization in real applications.

**Datasets of PLBA.** Existing PLBA datasets can be roughly divided into three categories. The first category includes datasets such as PDBbind, BindingMOAD [52] and CSAR-HiQ [53], which contain 3D co-crystal structures of protein–ligand complexes determined by structural characterization methods and experimentally determined binding affinity values. Such datasets have small yet high-quality data and are typically widely used for training structure-based DL models [54, 55]. For instance, the widely used PDBbind v2016 dataset contains 13 283 data carefully collected from Protein Data Bank (PDB) [56].

The second category comprises datasets such as ChEMBL [25] and BindingDB [26]. These datasets contain a large number of PLBA measurements. However, unlike the previous category datasets, the 3D co-crystal structures of the corresponding protein–ligand complexes are not provided. Instead, these datasets offer access to protein Uniprots and ligand SMILES representations. Despite the absence of structural information, these datasets are valuable for training affinity prediction models due to their extensive coverage of experimental binding affinity labels. The third category contains datasets where the 3D structure and binding affinity value of the protein–ligand complex calculated by molecular docking [57]. An example of such a dataset is the CrossDocked2020 dataset, containing about 22.5 million docked complex structures and affinity scores [58]. Due to the lack of experimental affinity labels, these datasets are often used to train generative models rather than affinity prediction models [59].

**Pre-training for biomolecules.** Much effort has been devoted to biomolecular pre-training to achieve better performance on related tasks. For small molecules and proteins, a series of self-supervised pre-training methods based on molecular graphs [17–20, 60] and protein sequences [21–24, 61, 62] have been proposed, respectively. However, these existing pre-training methods are designed for individual molecules [63], and there is still a gap in the research on pre-training methods for protein–ligand affinity.

**Pairwise learning to rank.** Learning to Rank (LTR) is an essential research topic in many areas, such as information retrieval and recommendation systems [64–66]. The common solutions of LTR could be basically categorized into three types: pointwise, pairwise and listwise. Among these methods, pairwise LTR models are widely used in practice due to their efficiency and effectiveness. These years have witnessed the success of pairwise methods, such as BPR [67], RankNet [68], GBRank [69] and RankSVM [70]. In addition, recent studies have shown that the bias between labels can be effectively solved using pairwise methods [30]. In previous PLBA prediction research, where models are directly trained on the clean and high-quality dataset PDBbind, LTR is not necessary. In this work, we define the bioassay noise problem and propose a strategy to leverage LTR techniques to overcome it.

## MULTI-TASK BIOASSAY PRE-TRAINING

In this section, we formalize the problem of pre-training for PLBA and then introduce our proposed MBP framework.

**Problem formulation.** Conceptually, given a protein $P$, a ligand $L$ and the binding conformation $C$ of the ligand to the protein, the problem of structure-based PLBA prediction is to learn a model $f(P, L, C)$ to predict the binding affinity. However, due to the rarity and high cost of ground truth 3D structure data, the training of structure-based PLBA prediction models has to be restricted to PDBbind with co-crystal structures. In this work, we aim to leverage the ChEMBL dataset, which contains large-scale PLBA data but without 3D structures. As discussed in Introduction section, in order to pre-train a PLBA prediction model on ChEMBL, we have to resolve three challenges, namely label variety, label noise and missing conformation.

### Framework overview

**Figure 2:**
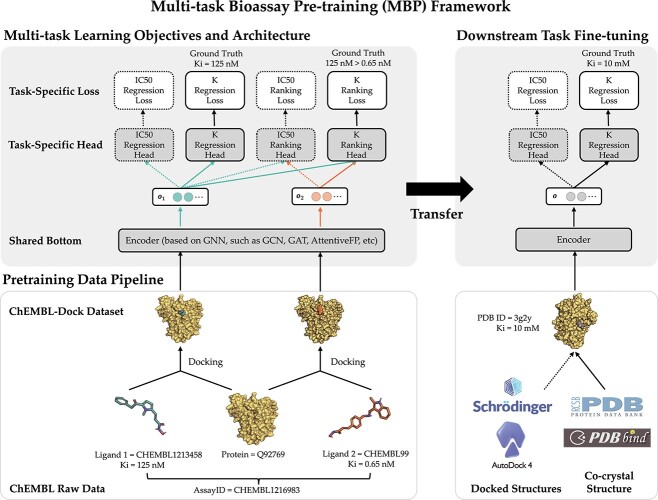
The framework of MBP in pre-training and fine-tuning. The solid arrows indicate the flow path of the running examples of AssayID = CHEMBL1216983 during pre-training and PDB ID = 3g2y during fine-tuning.

The framework overview of MBP is illustrated in Figure 2, which includes three main parts: (i) pre-training data pipeline; (ii) multi-task learning objectives and architecture and (iii) downstream task fine-tuning. In this section, we describe them in detail.

**Pre-training data pipeline.** Before introducing the pre-training data pipeline, we provide the necessary definitions of a bioassay and a bioassay-specific data pair in MBP.

Definition 1. (A Bioassay in MBP) A bioassay is defined as an analytical method to determine the concentration or potency of a substance by its effect on living animals or plants (in vivo) or on living cells or tissues (in vitro) [71]. In this work, we mainly focus on bioassays measuring *in vitro* binding of ligands to a protein target. Formally, the $i$th bioassay is denoted as

(1) \begin{align*}& A_i = \left(P_i, \left\{(L_{ij}, y_{ij})\right\}_{j=1}^{n_i}, t_i\right).\end{align*}

It means that there are $n_i$ experimental records in bioassay $A_i$, and each record measures the binding affinity $y_{ij}$ of a ligand $L_{ij}$ to the protein target $P_i$. And the type of binding affinity in bioassay $A_i$ is $t_i$; in this work, we consider $t_i\in \{{\text{Ki}}, {\text{Kd}}, {\text{IC50}}\}$. The ChEMBL dataset can be formalized as a collection of bioassays such that $\mathcal{D} = (A_1, A_2, \cdots )$.

Definition 2. (A bioassay-specific data pair) A bioassay-specific data pair is a six-tuple $(P_i, L_{ij}, L_{ik}, y_{ij}, y_{ik}, t_i)$, indicating that there is a bioassay $A_i$, which includes the binding measurement of ligand $L_{ij}$ and ligand $L_{ik}$ to a protein target $P_i$. And the experimentally measured binding affinity (with type $t_i$) is $y_{ij}$ and $y_{ik}$, respectively.

The bioassay-specific data pairs in this work are extracted and randomly sampled from ChEMBL bioassays [25]. We first sample a bioassay $A_i$ with probability proportional to its size, i.e. ${\text{Prob}}(A_i) \propto n_i$. Then, we randomly pick two different ligands $L_{ij}$ and $L_{ik}$ from the sampled assay $A_i$, together with their binding affinity $y_{ij}$ and $y_{ik}$. The above sampling process produces a bioassay-specific data pair $(P_i, L_{ij}, L_{ik}, y_{ij}, y_{ik}, t_i)$ as defined in Definition 2. Taking the running case shown in Figure 2 as an example, the data pair from a ChEMBL bioassay (with AssayId = CHEMBL1216983) can be written as (Q92769, CHEMBL1213458, CHEMBL99, 125nM, 0.65nM, Ki).

Knowledge of the binding conformation of a ligand to a target protein plays a vital role in structure-based drug design, particularly in predicting binding affinity. However, the ground truth co-crystal structure of a protein–ligand complex is experimentally very expensive to determine and is therefore not available in ChEMBL. Consequently, we only have the binding affinities (e.g. $y_{ij}$ and $y_{ik}$) of a ligand to a protein without knowing their conformation and relative orientation. To solve the above missing data problem, we propose to use computationally determined docking poses as an approximation to the true binding conformations. Specifically, we construct a large-scale docking dataset named ChEMBL-Dock from ChEMBL. For each protein–ligand pair in ChEMBL, we generate its docking poses according to the following three steps. Firstly, we use RDKit library [72] to generate 3D conformations from the 2D SMILES of the ligand. Then, the 3D structure of a protein is extracted from PDBbind according to its UniProt ID. Finally, we use docking software SMINA [73] to generate the docking poses of the protein–ligand pair. Throughout the rest of this paper, we denote the docking conformation of protein $P_i$ and ligand $L_{ij}$ as $C_{ij}$. The detailed data curation process of ChEMBL-Dock can be found later in the experiments section.

Overall, the pre-training data pipeline generates bioassay-specific data pairs $(P_i, L_{ij}, L_{ik}, y_{ij}, y_{ik}, t_i)$, retrieves their docking conformations—$C_{ij}$ and $C_{ik}$—from the pre-processed ChEMBL-Dock datasets, and then feeds them into a multi-task learning model which we will discuss below.

**Figure 3:**
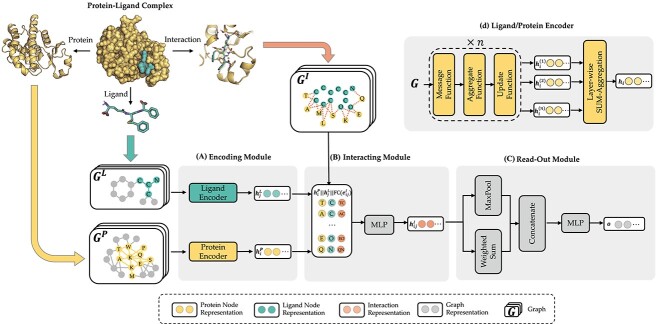
Shared bottom encoder of MBP. It contains three modules: **(A**) encoding module, (**B**) interacting module and (**C**) read-out module. (**D**) The detailed GNN model of the ligand/protein encoder in the encoding module.

**Multi-task learning objectives and architecture.** As discussed before, there are three main challenges of applying pre-training to the PLBA problem—missing conformation, label variety and label noise. In this section, we propose to solve the label variety and label noise problem via multi-task learning.

For the label variety challenge, it is intuitive and straightforward to introduce label-specific tasks for each type of binding affinity measurement. In this work, we define two categories of label-specific tasks—IC50 task and K={Ki, Kd} task, which handle bioassay data with affinity measurement type IC50 and Ki/Kd, respectively. Here, we merge Ki and Kd as a single task following [74], and the main reasons are 2-fold. Firstly, Ki and Kd are calculated in the same way, except that Kd only considers the physical binding, while Ki specifies the biological effect of this binding to be inhibition. Therefore, they can essentially be seen as the same label type. Secondly, the number of Kd data is significantly less compared with Ki data, which may lead to data imbalance if we were to design a separate Kd task.

For the label noise challenge, instead of leveraging learning with noisy labels techniques [75], we turn to utilize the intrinsic characteristics of bioassay data. Generally, the label noise challenge in ChEMBL stems mainly from its data sources and curation process. The binding affinity values from different bioassays were measured under various experimental protocols and conditions (such as temperature and pH value), leading to systematic errors between different assays. However, the binding affinity labels within the same bioassay were usually determined under similar experimental conditions. Thus, intra-bioassay data are more consistent than inter-bioassay ones, and the comparison within a bioassay is much more meaningful. Inspired by the above characteristics of bioassay data, we design both regression tasks and ranking tasks in MBP. To be more formal, given a bioassay-specific data pair $(P_i, L_{ij}, L_{ik}, y_{ij}, y_{ik}, t_i)$, the regression task is to directly predict the binding affinity $y_{ij}$, while the ranking task is to compare the binding affinity values within the bioassay, i.e. to classify whether $y_{ij} < y_{ik}$ or $y_{ij}> y_{ik}$.

In summary, we have $2\times 2=4$ tasks in MBP, namely the IC50 regression task, IC50 ranking task, K regression task and K ranking tasks. An illustration of these tasks can be found in Figure 2.

As for the multi-task learning architecture, we adopt the shared-bottom technique (also known as the hard parameter sharing) [76] in MBP. Such a technique shares a bottom encoder among all tasks while keeping several task-specific heads. As illustrated in Figure 2, the model architecture consists of a shared encoder network $f_{\text{Enc}}$ and four task specific heads—an IC50 regression head $f_{\text{IC50, Reg}}$, a K={Ki,Kd} regression head $f_{\text{K, Reg}}$, an IC50 ranking head $f_{\text{IC50, Rank}}$ and a K={Ki,Kd} ranking head $f_{\text{K, Rank}}$. Given a bioassay-specific data pair $(P_i, L_{ij}, L_{ik}, y_{ij}, y_{ik}, t_i)$ together with their conformation $C_{ij}$ and $C_{ik}$ from the pre-training data pipeline, the shared bottom encoder maps them into compact hidden representations shared among tasks 


(2) \begin{align*}& {\boldsymbol{o}}_1 = f_{\text{Enc}}(P_i, L_{ij}, C_{ij})\ \text{and}\ {\boldsymbol{o}}_2 = f_{\text{Enc}}(P_i, L_{ik}, C_{ik}).\end{align*}

There are many possibilities for implementing an encoder for protein–ligand complexes, including but not limited to models based on 3D-CNN [15, 58, 77], GNN [11, 12, 78] and Transformer [10, 79, 80]. In MBP, we propose a simple and effective shared bottom encoder. For the sake of clarity, we defer its implementation detail later and focus on multi-task learning in this section.

For the regression task, we pick the task-specific regression head $f_{t_i,\text{Reg}}$ according to the label type $t_i \in \{\text{IC50}, \text{K}\}$ (recall that Ki and Kd have been merged to be a single label type K) and predict the binding affinity to be $\hat{y}_{ij} = f_{t_i,\text{Reg}}({\boldsymbol{o}}_1)$. The regression loss is calculated using the mean squared error (MSE) loss between ground truth $y_{ij}$ and the predicted value $\hat{y}_{ij}$. More formally, the regression loss is defined as 


(3) \begin{align*}& L_{\text{Reg}} = \text{MSE} \left(\hat{y}_{ij}, y_{ij}\right).\end{align*}

It is worth mentioning that for a data pair, only label $y_{ij}$ will be used to compute the regression loss.

Similarly, for the ranking task, we select the task-specific ranking head $f_{t_i,\text{Rank}}$ according to the label type $t_i \in \{\text{IC50}, \text{K}\}$, concatenate the hidden representations as ${\boldsymbol{o}}_1 || {\boldsymbol{o}}_2$ and then predict the pairwise ranking to be $\hat{r}_{ijk} = f_{t_i,\text{Rank}}({\boldsymbol{o}}_1 || {\boldsymbol{o}}_2)$. The ranking loss is calculated as the binary cross entropy loss between ground truth $\mathbb{I}[y_{ij}> y_{ik}]$ and the predicted value $\hat{r}_{ijk}$, where $\mathbb{I}(\cdot )$ denotes the indicator function. More formally, the ranking loss is defined as 


(4) \begin{align*}& L_{\text{Rank}} = \text{BCE}\left(\hat{r}_{ijk}, \mathbb{I}[y_{ij}> y_{ik}]\right).\end{align*}

The overall loss function for a bioassay-specific data pair is a weighted sum of the regression loss in Equation 3 and ranking loss in Equation 4


(5) \begin{align*}& L_{\text{MBP}} = L_{\text{Rank}} + \lambda \times L_{\text{Reg}},\end{align*}

where $\lambda $ is the weight coefficient for regression loss.

Overall, we introduce multi-task learning into MBP, aiming to deal with label variety and label noise problems. In the illustrative example of MBP shown in Figure 2, MBP accepts the bioassay-specific data pair (Q92769, CHEMBL1213458, CHEMBL99, 125nM, 0.65nM, Ki) and their docking poses as inputs, encodes them to hidden representations, forwards the K regression head to predict $y_{ij}=125$nM and also forwards the K ranking head to classify $\text{125nM}> \text{0.65nM}$.

**Downstream task fine-tuning.** The final part of the MBP framework is the downstream task fine-tuning. Given the 3D structure of a protein–ligand complex as input, the downstream task is to predict its binding affinity. The 3D structure can be either an experimentally determined co-crystal structure or a computationally determined docking pose. We transfer and fine-tune the shared bottom encoder $f_{\text{Enc}}$ together with the regression heads $f_{\text{IC50, Reg}}$ and $f_{\text{K, Reg}}$ in downstream PLBA datasets (such as PDBbind). The right panel of Figure 2 shows how the transferred model predicts the Ki value for a protein–ligand complex from PDBbind (PDB ID=3g2y).

### Shared bottom encoder

For large-scale pre-training, a simple and effective backbone model is of utmost importance. Thus, we design the shared bottom encoder based only on vanilla GNN models. To simplify, we assume that the input of the shared bottom encoder is a 3-tuple $(P, L, C)$, indicating a protein $P$, a ligand $L$ and their binding conformation $C$.

**Representing protein-ligand complex as multi-graphs.** The input protein–ligand complex $(P, L, C)$ is processed into three graphs—a ligand graph, a protein graph and a protein–ligand interaction graph. We formally define the three graphs as follows.

Definition 3. (Ligand graph) A ligand graph, denoted by $\mathcal{G}^L=(\mathcal{V}^L, \mathcal{E}^L)$, is constructed from the input ligand $L$. $\mathcal{V}^L$ is the node set where node $i$ represents the $i$th atom in the ligand. Each node $i$ is also associated with (i) atom coordinate $c^L_i$ retrieved from the binding conformation $C$ and (ii) atom feature vector ${\boldsymbol{x}}^L_i$. The edge set $\mathcal{E}^L$ is constructed according to the spatial distances among atoms. More formally, the edge set is defined to be

(6) \begin{align*}& \mathcal{E}^L = \left\{(i,j): \left\lVert c^L_i - c^L_j\right\rVert_{2}< cut^{L}, \forall i, j \in \mathcal{V}^L\right\},\end{align*}

where $cut^{L}$ is a distance threshold, and each edge $(i,j)\in \mathcal{E}^L$ is associated with an edge feature vector ${\boldsymbol{e}}^L_{ij}$. The node and edge features are obtained by Open Babel [81].

Definition 4. (Protein graph) A protein graph, denoted by $\mathcal{G}^P=(\mathcal{V}^P, \mathcal{E}^P)$, is constructed from the input protein $P$. $\mathcal{V}^P$ is the node set where the node $i$ represents the $i$th residue in the protein. Each node $v^P_i$ is also associated with (i) the alpha carbon coordinate of the $i$th residue $c^P_i$ retrieved from the binding conformation $C$ and (ii) the residue feature vector ${\boldsymbol{x}}^P_i$. The edge set $\mathcal{E}^P$ is constructed according to the spatial distances among atoms. More formally, the edge set is defined to be

(7) \begin{align*}& \mathcal{E}^P = \left\{(i,j): \left\lVert c^P_i - c^P_j\right\rVert_{2}< cut^{P}, \forall i, j \in \mathcal{V}^P \right\},\end{align*}

where $cut^{P}$ is a distance threshold, and each edge $(i,j)\in \mathcal{E}^P$ is associated with an edge feature vector ${\boldsymbol{e}}^P_{ij}$. The node and edge features are obtained following [82].

Definition 5. (Interaction graph) The protein–ligand interaction graph $\mathcal{G}^{I}=( \mathcal{V}^{P}, \mathcal{V}^{L}, \mathcal{E}^{I})$ is a bipartite graph constructed based on the protein–ligand complex, whose nodes set are the union of protein residues $\mathcal{V}^{P}$ and ligand atoms $\mathcal{V}^{L}$. The edge set $\mathcal{E}^{I}$ models the protein–ligand interactions according to spatial distances. More formally,

(8) \begin{align*}& \mathcal{E}^{I} = \left\{(i,j): \left\lVert c^P_i - c^L_j\right\rVert_{2} < cut^I, \forall i \in \mathcal{V}^P, j \in \mathcal{V}^L \right\},\end{align*}

where $cut^{I}$ is a spatial distance threshold for interaction, and each edge $(i,j)\in \mathcal{E}^I$ is associated with an edge feature vector ${\boldsymbol{e}}^I_{ij}$. The edge features are obtained following [82].

When constructing multi-graphs, we follow previous work [12, 55, 83–87] and set the distance thresholds as $cut^L=5.0{{\AA }}$, $cut^P=8.0{{\AA }}$ and $cut^{I}=12.0$Å, respectively. Limited by space, we defer the more detailed multi-graph generation pseudo-code to Appendix 2.

**Encoding module (ligand/protein encoder).** Having represented the protein–ligand complex as multi-graphs, we respectively feed the ligand graph $\mathcal{G}^L$ and the protein graph $\mathcal{G}^P$ into the ligand encoder and the protein encoder, aiming to extract informative node representations. More formally, taking $\mathcal{G}^L$ and $\mathcal{G}^P$ as inputs, we have 


(9) \begin{align*}& {\boldsymbol{H}}^L = \text{GNN}(\mathcal{G}^L)\ \text{and} \ {\boldsymbol{H}}^P = \text{GNN}(\mathcal{G}^P).\end{align*}

Here, ${\boldsymbol{H}}^L$ is the ligand embedding matrix of shape $\left \lvert \mathcal{V}^L\right \rvert \times d$. And the $i$th row of ${\boldsymbol{H}}^L$, denoted by ${\boldsymbol{h}}^L_i$, represents the embedding of the $i$th ligand atom. Similarly, ${\boldsymbol{H}}^P$ is the protein embedding matrix of shape $\left \lvert \mathcal{V}^P\right \rvert \times d$. And the $i$th row of ${\boldsymbol{H}}^P$, denoted by ${\boldsymbol{h}}^P_i$, represents the embedding of the $i$th protein residue.

Encoders used here can be any GNN model, such as GCN, GAT, GIN, EGNN, AttentiveFP, etc. Here, we briefly review GNNs following the message-passing paradigm following [88] and [89]. For simplicity and convenience, we assume that the GNN operates on graph $\mathcal{G}$ with node features ${\boldsymbol{x}}_i$ and edge features ${\boldsymbol{e}}_{ij}$, and temporarily ignore whether it is a ligand or protein graph. The message-passing process runs for several iterations. At the $\ell $th iteration, the message-passing is defined according to a message function $M_\ell $, an aggregation function $\text{AGGREGATOR}_\ell $ and an update function $U_\ell $. The embedding ${\boldsymbol{h}}_i^{(\ell )}$ of node $i$ is updated via its message ${\boldsymbol{m}}_i^{(\ell +1)}$


(10) \begin{align*} {\boldsymbol{m}}^{(\ell+1)}_i &= \text{AGGREGATOR}_\ell\left(\left\{M_\ell\left({\boldsymbol{h}}^{(\ell)}_i,{\boldsymbol{h}}^{(\ell)}_j, {\boldsymbol{e}}_{ij}\right), j \in \mathcal{N}(i)\right\}\right),\nonumber\\{\boldsymbol{h}}^{(\ell+1)}_i&= U_\ell \left({\boldsymbol{h}}^{(\ell)}_i, {\boldsymbol{m}}^{(\ell+1)}_i\right), \end{align*}

where $\mathcal{N}(i)$ is the neighbors of node $i$. Finally, after $n$ iterations of message passing, we sum up the node representations of each layer to get the final node representation, i.e. ${\boldsymbol{h}}_i = \sum _{\ell =1}^{n}{{\boldsymbol{h}}^{(\ell )}_i}$. The GNNs with layer-wise aggregation are also known as jumping knowledge networks [90].

**Interacting module.** After extracting ligand atom embedding ${\boldsymbol{h}}^L_i$ and protein residue embedding ${\boldsymbol{h}}^P_j$ from the encoding module, the interacting module is designed to conduct knowledge fusion according to the protein–ligand interaction graph. For each protein–ligand interaction edge $(i,j) \in \mathcal{E}^I$, we define its interaction embedding as the concatenation of the protein residue embedding ${\boldsymbol{h}}^P_i$, ligand atom embedding ${\boldsymbol{h}}^L_j$ and transformed edge features. More formally, 


(11) \begin{align*}& {\boldsymbol{h}}^I_{ij} = \text{MLP}\left({\boldsymbol{h}}^P_i || {\boldsymbol{h}}^L_j || \text{FC}({\boldsymbol{e}}^I_{ij})\right),\end{align*}

where || is the concatenation operator, MLP is a multilayer perceptron and FC is a fully connected layer.

**Read-out module.** After obtaining interaction embeddings ${\boldsymbol{h}}^I_{ij}$ for each protein–ligand interaction edge $(i, j) \in \mathcal{E}^{I}$, we further apply an attention-based weighted sum operation to read out a global embedding for the whole protein–ligand complex 


(12) \begin{align*}& {\boldsymbol{o}}_{\text{sum}} = \sum_{(i,j) \in \mathcal{E}^I} \text{tanh}({\boldsymbol{w}}^\top{\boldsymbol{h}}^I_{ij}){\boldsymbol{h}}^I_{ij},\end{align*}

where ${\boldsymbol{w}}$ is the attention vector and $\text{tanh}$ is the hyperbolic tangent function. Besides, a global maximum pooling operation is adopted to highlight the most informative interaction embedding, s.t., ${\boldsymbol{o}}_{\max } = \text{MaxPool}({\boldsymbol{h}}^I_{ij})$. We concatenate the above two graph-level embedding to form the final graph embedding for the protein–ligand complex $(P, L, C)$, i.e. ${\boldsymbol{o}} = {\boldsymbol{o}}_{\text{sum}} || {\boldsymbol{o}}_{\max }$.

## EXPERIMENTS

### Pre-training dataset: ChEMBL-Dock

**Figure B1:**
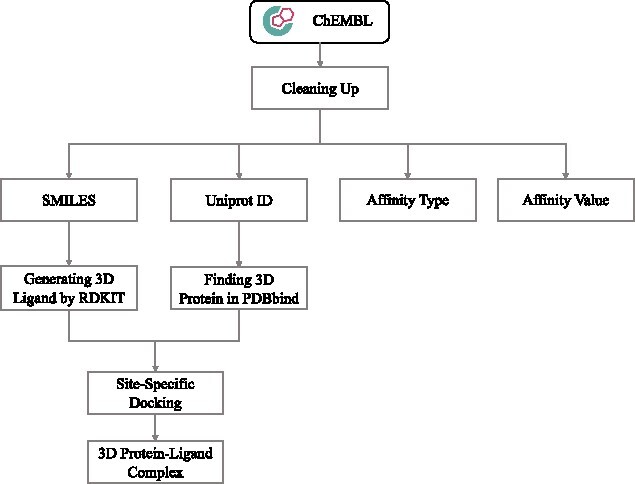
Construction process of ChEMBL-Dock.

In this section, we formally introduce ChEMBL-Dock as the pre-training dataset of MBP. We first describe its construction process, including the data collection & cleaning step and molecular docking settings, and then compare it to other existing PLBA datasets. The detailed ChEMBL-based curation workflow can be found in Figure B1 in Appendix 2.

**Data collection and cleaning.** We first clean up the database to select high-quality PLBA data, as the bioactivity database ChEMBL covers a broad range of data, including binding, functional, absorption, distribution, metabolism and excretion data. The filtering criterias in ChEMBL we used are

- STANDARD_TYPE = ‘IC50’/‘Ki’/‘Kd’ (other types of affinity, such as EC50, have limited bioassay data in ChEMBL);
- STANDARD_RELATION = ‘=’;
- STANDARD_UNITS = ‘nM’;
- ASSAY_TYPE = ‘B’ (meaning that the data is binding data);
- TARGET_TYPE = ‘SINGLE PROTEIN’;
- COMPONENT_TYPE = ‘PROTEIN’;
- MOLECULE_TYPE = ‘Small molecule’;
- BAO_FORMAT = ‘BAO_0000357’ (meaning that in the assays, only results with single protein format were considered).

Besides the above filters, we further exclude bioassays with only one protein–ligand pair and bioassays with more than one affinity type. We then prepare the 3D structures of proteins and ligands, respectively. The 3D structures of proteins are extracted from PDBbind using their Uniprot ID, while the 3D structures of ligands are generated using RDKit. We ignore all proteins not in PDBbind and ligands that RDkit fails to generate a conformation. The final dataset comprises 313 224 protein–ligand pairs from 21 686 bioassays, containing a total of 231 948 IC50 protein–ligand pairs from 14 954 bioassays, 69 127 Ki protein–ligand pairs from 5397 bioassays and 12 149 Kd protein–ligand pairs from 1335 bioassays, respectively.

**Molecular docking.** Molecular docking software SMINA [73], which is a version of AutoDock Vina with a custom empirical SF, is utilized to generate 3D protein–ligand complexes. As the proteins of these data have already been included in the PDBbind database, we specify a search space to perform site-specific docking. The search space is a $22.5{{\AA }} \times 22.5{{\AA }} \times 22.5{{\AA }}$ grid box centered on the ligand of the PDBbind complex which has the same proteins. For these data, we can accurately identify the binding sites of the protein–ligand complex from the 3D structure information provided in the PDBbind database. The docking parameters ’exhaustiveness’ and ’seed’ are set to 8 and 2022, respectively. SMINA outputs 9 candidate poses for each protein–ligand pair, resulting in 2 819 016 poses for 313 224 protein–ligand pairs together. In this work, we use the top-1 poses for pre-training. Additionally, to demonstrate the reliability of these structures, we conduct a quality evaluation of the top-1 poses in Appendix 4. These results provide evidence that the computationally generated structures possess reasonably good quality, rendering them suitable for use.

**Figure 4:**
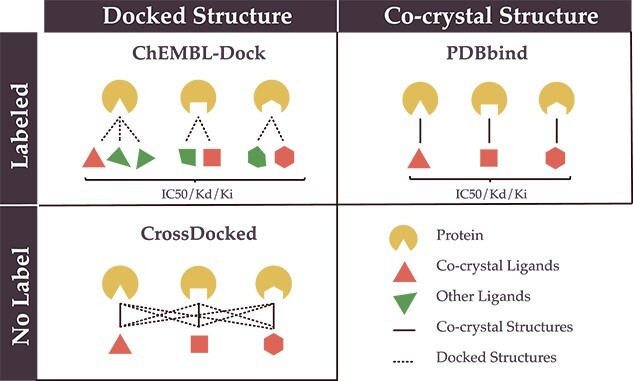
Comparison of ChEMBL-Dock with PDBbind and CrossDocked on label and structure.

**Comparison to other PLBA datasets.** To gain a better understanding of the advantages of our dataset, we compare ChEMBL-Dock with other related datasets in terms of the label, 3D structure, protein diversity, molecular diversity and dataset size in Figure 4 and Table 1. Here, the diversity of proteins and ligands is characterized by the number of unique ligand canonical SMILES representations and protein Uniprot IDs. Combining the strengths of molecular docking and ChEMBL, ChEMBL-Dock provides a large-scale 3D protein–ligand complex dataset with corresponding experimental affinity labels. While the quality of the 3D structures of the complexes in ChEMBL-Dock is not as high as that of the 3D co-crystal structures of the protein–ligand complexes in PDBbind, ChEMBL-Dock provides a much larger number of 3D structures of protein–ligand complexes than the PDBbind database. By comparing ChEMBL-Dock and CrossDocked, two datasets generated through molecular docking, it is evident that ChEMBL-Dock exhibits a higher molecular diversity than CrossDocked, suggesting its potential to provide a more comprehensive dataset for drug discovery research.

**Table 1:** Overview of data represented in PDBbind, CrossDocked and ChEMBL-Dock, respectively

	**PDBbind**	**CrossDocked**	**ChEMBL-Dock**
Protein	3890	2922	963
Ligand	15 193	13 780	200 728
Protein-ligand pair	19 443	/	313 224
Pose	19 443	22 584 102	2 819 016
Bioassay	/	/	21 686

### Experimental setup

**Downstream datasets.** Two publicly available datasets are used to comprehensively evaluate the performance of models.

- **PDBbind v2016** [16] is a famous benchmark for evaluating the performance of models in predicting PLBA. The dataset includes three overlapping subsets: the general set (13 283 3D protein–ligand complexes), the refined set (4057 complexes selected out of the general set with better quality) and the core set (285 complexes selected as the highest quality benchmark for testing). We refer to the difference between the refined and core subsets as the refined set for convenience. The general set contains IC50 data and K data, while the refined set and core only contain K data. In this paper, the core set is used as the test set, and we train models on the refined set or the general set.
- **CSAR-HiQ** [53] is a publicly available dataset of 3D protein–ligand complexes with associated experimental affinity labels. Data included in CSAR-HiQ are K data. When training models on the refined set of PDBbind, CSAR-HiQ is typically used to evaluate the generalization performance of the model [12]. In this paper, we create an independent test set of 135 samples based on CSAR-HiQ by removing samples that already exist in the PDBbind v2016 refined set.

**Baselines.** We first compare MBP with four families of methods. The first family is machine learning-based methods such as Linear Regression, Support Vector Regression and RF-Score [45]. The second family is CNN-based methods, including Pafnucy [8] and OnionNet [9]. The third family of baselines is GraphDTA [48] methods, including GCN, GAT, GIN and GAT-GCN. The fourth family of baselines is GNN-based methods containing SGCN [49], GNN-DTI [91], DimeNet [50], CMPNN [51] and SIGN [12]. These models are re-trained and tested on the same training set and the testing set follows SIGN’s setting.

In addition, for recent proposed powerful DL-based affinity prediction methods, including PLIG [92], PaxNet [93], GLI [94], KIDA [95], GraphscoreDTA [96], PLANET [97], GIGN [98], molecular docking method TANKBind [54] and molecular pre-trained method Transformer-M [60], we directly use their reported results and ignoring the difference of their training settings.

**Evaluation metrics.** Root Mean Square Error (RMSE), Mean Absolute Error (MAE), Standard Deviation (SD) and Pearson’s correlation coefficient (R) are used to evaluate the performance of PLBA prediction [12]. The definition of these metrics can be found in Appendix 3.

**Training parameter settings.** The models were trained using Adam [99] with an initial learning rate of $10^{-3}$ and an $L_{2}$ regularization factor of $10^{-6}$. The learning rate was scaled down by 0.6 if no drop in training loss was observed for 10 consecutive epochs. The dropout value was set to 0.1 and batch size was set to 256 and 128 for pre-training and fine-tuning, respectively. For pre-training, the number of training epochs was set to 100, while for fine-tuning, the number of training epochs was set to 1000 with an early stopping rule of 70 epochs if no improvement in the validation performance was observed. More details about model hyperparameters are in Appendix 3.

### Experimental results

In this work, we employ five different GNNs in the shared bottom encoder of MBP, which are denoted as MBP-X (where X corresponds to the GNN used) for distinction. For example, MBP-GCN denotes the MBP model using GCN in its shared bottom encoder. Unless specified otherwise, AttentiveFP is used as the default GNN in MBP.

**Overall performance comparison.** We first fine-tune MBP on the PDBbind refined set and report the test performance averaged over five repetitions for each method on the PDBbind core set in Table 2. Here, all methods are trained on the PDBbind v2016 refined set. To assess statistical significance, we employ two-tailed $t$-tests to determine if there is a significant performance difference between MBP and other baselines. Test results are also shown in Table 2. It can be observed that MBP achieves the best performance across all metrics of the two publicly available datasets. In particular, MBP-AttentiveFP and MBP-EGNN outperforms all competing methods on the PDBbind core set. Compared with SIGN, MBP-AttentiveFP achieving an improvement of 4.0, 2.7, 6.3 and 3.5% on RMSE, MAE, SD and R, respectively.

**Table 2:** Test performance comparison on the PDBbind v2016 core set and the CSAR-HiQ dataset. The mean RMSE, MAE, SD and R (std) over three repetitions are reported. The best results are highlighted in bold, and the second best results are underlined

Method		PDBbind core set				CSAR-HiQ dataset			
		RMSE($\downarrow $)	MAE($\downarrow $)	SD($\downarrow $)	R($\uparrow $)	RMSE($\downarrow $)	MAE($\downarrow $)	SD($\downarrow $)	R($\uparrow $)
ML-based	LR	1.675 (0.000) (*)	1.358 (0.000) (*)	1.612 (0.000) (*)	0.671 (0.000) (*)	2.071 (0.000) (*)	1.622 (0.000) (*)	1.973 (0.000) (*)	0.652 (0.000) (*)
	SVR	1.555 (0.000) (*)	1.264 (0.000) (*)	1.493 (0.000) (*)	0.727 (0.000) (*)	1.995 (0.000) (*)	1.553 (0.000) (*)	1.911 (0.000) (*)	0.679 (0.000) (*)
	RF-Score	1.446 (0.008) (*)	1.161 (0.007) (*)	1.335 (0.010) (*)	0.789 (0.003) (*)	1.947 (0.012) (*)	1.466 (0.009) (*)	1.796 (0.020) (*)	0.723 (0.007) (*)
CNN-based	Pafnucy	1.585 (0.013) (*)	1.284 (0.021) (*)	1.563 (0.022) (*)	0.695 (0.011) (*)	1.939 (0.103) (*)	1.562 (0.094) (*)	1.885 (0.071) (*)	0.686 (0.027) (*)
	OnionNet	1.407 (0.034) (*)	1.078 (0.028) (*)	1.391 (0.038) (*)	0.768 (0.014) (*)	1.927 (0.071) (*)	1.471 (0.031) (*)	1.877 (0.097) (*)	0.690 (0.040) (*)
GraphDTA	GCN	1.735 (0.034) (*)	1.343 (0.037) (*)	1.719 (0.027) (*)	0.613 (0.016) (*)	2.324 (0.079) (*)	1.732 (0.065) (*)	2.302 (0.061) (*)	0.464 (0.047) (*)
	GAT	1.765 (0.026) (*)	1.354 (0.033) (*)	1.740 (0.027) (*)	0.601 (0.016) (*)	2.213 (0.053) (*)	1.651 (0.061) (*)	2.215 (0.050) (*)	0.524 (0.032) (*)
	GIN	1.640 (0.044) (*)	1.261 (0.044) (*)	1.621 (0.036) (*)	0.667 (0.018) (*)	2.158 (0.074) (*)	1.624 (0.058) (*)	2.156 (0.088) (*)	0.558 (0.047) (*)
	GAT-GCN	1.562 (0.022) (*)	1.191 (0.016) (*)	1.558 (0.018) (*)	0.697 (0.008) (*)	1.980 (0.055) (*)	1.493 (0.046) (*)	1.969 (0.057) (*)	0.653 (0.026) (*)
GNN-based	SGCN	1.583 (0.033) (*)	1.250 (0.036) (*)	1.582 (0.320) (*)	0.686 (0.015) (*)	1.902 (0.063) (*)	1.472 (0.067) (*)	1.891 (0.077) (*)	0.686 (0.030) (*)
	GNN-DTI	1.492 (0.025) (*)	1.192 (0.032) (*)	1.471 (0.051) (*)	0.736 (0.021) (*)	1.972 (0.061) (*)	1.547 (0.058) (*)	1.834 (0.090) (*)	0.709 (0.035) (*)
	DimeNet	1.453 (0.027) (*)	1.138 (0.026) (*)	1.434 (0.023) (*)	0.752 (0.010) (*)	1.805 (0.036) (*)	1.338 (0.026) (*)	1.798 (0.027) (*)	0.723 (0.010) (*)
	CMPNN	1.408 (0.028) (*)	1.117 (0.031) (*)	1.399 (0.025) (*)	0.765 (0.009) (*)	1.839 (0.096) (*)	1.411 (0.064) (*)	1.767 (0.103) (*)	0.730 (0.052) (*)
	SIGN	1.316 (0.031) (**)	1.027 (0.025)	1.312 (0.035) (*)	0.797 (0.012) (*)	1.735 (0.031) (*)	1.327 (0.040) (*)	1.709 (0.044) (*)	0.754 (0.014) (*)
**Ours**	MBP-GCN	1.333 (0.019)	1.056 (0.011)	1.306 (0.018)	0.800 (0.006)	1.718 (0.044)	1.348 (0.025)	1.659 (0.048)	0.751 (0.017)
	MBP-GIN	1.375 (0.031)	1.088 (0.026)	1.352 (0.044)	0.783 (0.016)	1.748 (0.028)	1.391 (0.026)	1.664 (0.050)	0.749 (0.017)
	MBP-GAT	1.393 (0.017)	1.122 (0.007)	1.367 (0.023)	0.778 (0.008)	1.703 (0.039)	1.317 (0.041)	1.606 (0.022)	0.769 (0.007)
	MBP-EGNN	1.298 (0.029)	1.023 (0.025)	1.262 (0.033)	0.815 (0.011)	1.649 (0.045)	1.242 (0.036)	1.548 (0.055)	0.788 (0.016)
	MBP-AttentiveFP	**1.263 (0.023)**	**0.999 (0.024)**	**1.229 (0.026)**	**0.825 (0.008)**	**1.624 (0.037)**	**1.240 (0.038)**	**1.536 (0.052)**	**0.791 (0.016)**

^1^Results of baseline methods were taken from [12]. ^2^Here, we perform two-tailed $t$-tests between MBP-AttentiveFP and other baselines on all metrics. We use * and ** to denote the P-value as less than 0.01 and 0.05, respectively.

Both MBP-EGNN and MBP-AttentiveFP surpass all baseline methods and are the best two methods in the current results.

**Figure 5:**
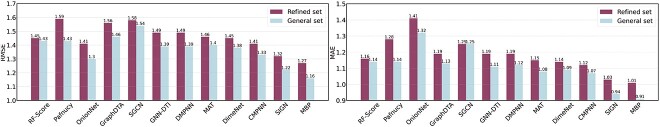
Performance improvements of baselines and MBP on the PDBbind benchmark when training on general set.

To further evaluate the generalization performance of the proposed model, we conduct an extra experiment on the PDBbind general set. As shown in Figure 5, comparing to all baselines, MBP still achieves the best performance in terms of both RMSE and MAE.

Then, for recently proposed powerful methods, we list their training settings, test settings and prediction accuracy achieved from their papers in Table 3 to make a fair comparison. Here, we report the performance of MBP trained on the PDBbind v2016 general set. It is worth noting that there are notable variations in the training settings employed by these newest methods. Compared with methods trained in the PDBbind v2016 refined set, these methods show better prediction accuracy. Although MBP was not trained on the largest dataset, it surpasses all other methods in terms of both RMSE and Pearson metrics. Compared with previous SOTA methods GIGN, it achieves an improvement of 2.6 and 1.8% on RMSE and Pearson. These results underscore the effectiveness and competitiveness of MBP, even when compared with these highly powerful methods.

**Table 3:** Training setting, testing setting and test performance of recently proposed binding affinity prediction models. The best results are highlighted in bold, and the second best results are underlined

Method	Year	Training set	Testing set	RMSE ($\downarrow $)	R ($\uparrow $)
IGN	2021	PDBbind v2016 general set ($N=8$298)	PDBbind v2016 core set ($N=$262)	1.220*	0.837**
SIGN	2021	PDBbind v2016 refined set ($N=$11 993)	PDBbind v2016 core set ($N=$290)	1.220*	–
PLIG	2022	PDBbind v2020 general set + PDBbind v2016 refined set ($N=$19 451)	PDBbind v2016 core set ($N=$285)	1.210*	0.840**
PaxNet	2022	PDBbind v2016 refined set ($N=3$390)	PDBbind v2016 core set ($N=$290)	1.263*	0.815
GLI	2022	PDBbind v2016 refined set ($N=$3390)	PDBbind v2016 core set ($N=$290)	1.294*	–
TANKBind	2022	PDBbind v2020 general set ($N=$17 787)	PDBbind v2020 general set ($N=$363)	1.346*	0.736*
Transformer-M	2022	PDBbind v2016 refined set ($N=$3767)	PDBbind v2016 core set ($N=$290)	1.232*	0.830*
GIGN	2023	PDBbind v2019 general set ($N=$11 906)	PDBbind v2016 core set ($N=$285)	1.190**	0.840**
KIDA	2023	PDBbind v2016 general set ($N=$12 500)	PDBbind v2016 core set ($N=$285)	1.291*	0.837*
GraphscoreDTA	2023	PDBbind v2019 general set ($N=9$869)	PDBbind v2016 core set ($N=$279)	1.249*	0.831
PLANET	2023	PDBbind v2020 general set ($N=$15 616)	PDBbind v2016 core set ($N=$285)	1.247*	0.824*
MBP	2023	PDBbind v2016 general set ($N=11$ 906)	PDBbind v2016 core set ($N=$285)	**1.159**	**0.855**

^1^ All results of baseline methods are obtained from their papers to make a fair comparison. ^2^ Here, we perform two-tailed $t$-tests between MBP and other baselines. We use * and ** to denote the $P$-value as less than 0.01 and 0.05, respectively.

**Generalization performance comparison**. Besides prediction accuracy, generalization ability is important for affinity prediction models to be applied in real-world scenarios. To evaluate the model’s performance on new protein–ligand pairs, we first follow SIGN [12] to employ the CASR-HiQ dataset as an independent test set to evaluate the generalization ability in Table 2. By removing all CASR-HiQ samples that exist in the PDBbind v2016 refined set, we obtain a dataset containing 135 samples. We can observe that our model performs significantly better than other baselines in this dataset and settings. Particularly, it attains more than 6.3, 6.6, 10.1 and 4.9% on RMSE, MAE, SD and R gain compared with SIGN.

**Figure 6:**
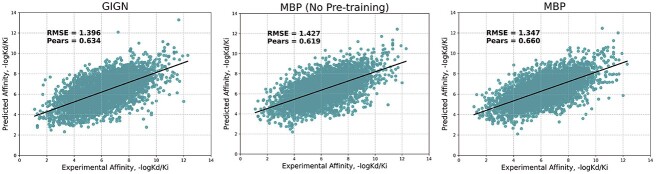
Performance of MBP on 4366 data that are unavailable in the PDBbind v2016 training set.

We further employ previous SOTA methods GIGN’s setting [98] to create a PDBbind 2019 holdout set, which contains 4366 data unavailable in PDBbind v2016 training set from PDBbind v2019. This temporal split setting presents a more challenging task and provides a more accurate reflection of the real-world scenario in drug discovery [100]. In Figure 6, we plot the prediction results of MBP, MBP without pre-traning and the previous SOTA method GIGN. On this difficult dataset, despite observing some performance degradation, MBP achieves an RMSE of 1.347, even outperforming many baselines’ performance in the PDBbind v2016 core set (Table 2). Additionally, MBP consistently outperforms GIGN, demonstrating its superior generalization ability. Without pre-training, MBP fails to surpass the performance of GIGN. These results highlight the enhanced generalization ability of MBP compared with other baselines and underscore the effectiveness of pre-training in improving generalization capability.

**Computation efficiency comparison.** In addition to prediction accuracy and generalization ability, we also conducted an efficiency comparison of MBP with several commonly used powerful models. Table 4 presents the training time and inference time on the PDBbind v2016 general set. Compared with previous methods, MBP requires an additional pre-training period, which takes $\sim $16 h. However, after pre-training, the fine-tuning and inference periods of MBP are efficient. It only takes about 5 s to infer 285 data points in the PDBbind v2016 core set (excluding data processing time). As shown in Table 4, although MBP requires more time for pre-training, the overall time is still acceptable ($\sim $21 h). Moreover, the trained model demonstrates sufficient efficiency to be applied in real-world inference scenarios. Overall, the results demonstrate that MBP not only achieves the best prediction accuracy and generalization ability but also exhibits no significant efficiency disadvantages compared with other methods.

**Table 4:** Training time and inferencing time on the PDBbind v2016 general set

Method	Time			
	Pre-training	Finetuning/Training	Inferencing	Total
OnionNet	–	about 1 h	about 1 s	about 1 h
SIGN	–	about 8 h	about 10 s	about 8 h
GIGN	–	about 1 h	about 1 s	about 1 h
MBP	about 16 h	about 4 h	about 5 s	about 21 h

### Ablation studies

In this section, we conduct extensive ablation studies to investigate the role of different components in MBP. All MBP models are fine-tuned on the PDBbind refined set.

**Multi-task learning objectives.** We perform an ablation study to investigate the effect of multi-task learning. Table 5 shows the results of our MBP with different learning tasks. We have two main observations:

- **Regarding regression tasks and ranking tasks**, we find that on both PDBbind core set and independent CSAR-HiQ set, MBP with a combination of both regression and ranking tasks can always outperform MBP with only regression or ranking tasks. And directly employing the ChEMBL-Dock data for regression pre-training may result in degradation of generalization ability. This observation indicates that the assembling of regression and bioassay-specific ranking contributes to the improvement of the model’s prediction accuracy and generalization ability.
- **Regarding IC50 tasks and K tasks**, we also find that MBP pre-trained with both IC50 and K tasks is better than that using only IC50 or K tasks. This implies that MBP is able to learn the task correlation between IC50 and K data from ChEMBL and transfer the knowledge to the PDBbind core set, which only contains Ki/Kd data.

**Table 5:** Ablation study of MBP with different pre-training tasks. The mean RMSE, MAE, SD and R (std) over five repetitions are reported. The best results are highlighted in bold, and the second best results are underlined

Regression		Ranking		PDBbind core set				CSAR-HiQ set			
IC50	K	IC50	K	RMSE$\downarrow $	MAE$\downarrow $	SD$\downarrow $	R$\uparrow $	RMSE$\downarrow $	MAE$\downarrow $	SD$\downarrow $	R$\uparrow $
				1.377 (0.045)	1.075 (0.040)	1.366 (0.042)	0.778 (0.015)	1.661 (0.028)	1.270 (0.035)	1.629 (0.043)	0.777 (0.008)
✓				1.364 (0.009)	1.077 (0.005)	1.351 (0.010)	0.784 (0.004)	1.693 (0.038)	1.293 (0.031)	1.628 (0.036)	0.761 (0.011)
		✓		1.418 (0.036)	1.120 (0.041)	1.398 (0.037)	0.766 (0.014)	1.787 (0.082)	1.386 (0.062)	1.756 (0.062)	0.714 (0.024)
✓		✓		1.315 (0.011)	1.055 (0.010)	1.268 (0.014)	0.813 (0.005)	1.690 (0.037)	1.268 (0.048)	**1.470 (0.052)**	0.764 (0.016)
	✓			1.292 (0.025)	1.018 (0.023)	1.267 (0.032)	0.813 (0.011)	1.704 (0.132)	1.254 (0.053)	1.586 (0.060)	0.746 (0.058)
			✓	1.372 (0.029)	1.096 (0.032)	1.365 (0.036)	0.779 (0.013)	1.659 (0.008)	1.294 (0.026)	1.601 (0.032)	0.771 (0.010)
	✓		✓	1.283 (0.023)	1.017 (0.014)	1.255 (0.032)	0.817 (0.010)	1.637 (0.018)	**1.233 (0.036)**	1.580 (0.008)	0.788 (0.011)
✓	✓			1.287 (0.025)	1.027 (0.024)	1.254 (0.031)	0.817 (0.010)	1.662 (0.048)	1.269 (0.032)	1.544 (0.058)	0.789 (0.018)
		✓	✓	1.325 (0.010)	1.048 (0.011)	1.307 (0.013)	0.800 (0.044)	1.674 (0.045)	1.293 (0.033)	1.616 (0.049)	0.766 (0.016)
✓	✓	✓	✓	**1.263 (0.023)**	**0.999 (0.024)**	**1.229 (0.026)**	**0.825 (0.008)**	**1.624 (0.037)**	1.240 (0.038)	1.536 (0.052)	**0.791 (0.016)**

These results justify the effectiveness of the multi-task learning objectives designed in MBP.

**GNN used in shared bottom encoder.** As shown in Table 2, we benchmark and compare MBP with different GNNs in the shared bottom encoder. We choose five popular GNN models—GCN, GIN, GAT, EGNN and AttentiveFP. EGNN and AttentiveFP are able to capture the 3D structure of biomolecules, while GCN, GIN and GAT are mainly designed for general graphs which cannot capture structural information directly. We have two interesting observations. Firstly, all GNN models, even the vanilla GCN, achieve comparable or better performance than previous methods. For example, MBP-GCN achieves an RMSE of 1.718 on the CSAR-HiQ set, slightly better than SIGN’s 1.735. Secondly, GNNs that explicitly capture 3D structure information (EGNN and AttentiveFP) outperform GNNs designed for general graphs (GCN, GAT, GIN).

**Figure 7:**
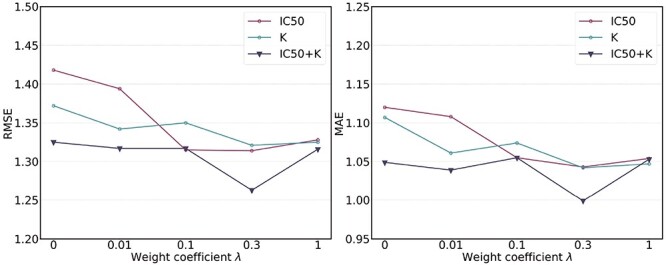
Test RMSE and MAE of MBP on the PDBbind core set with varying weight coefficients $\lambda $ of the regression loss.

**Hyper-parameter studies.** We ablate the weight coefficient $\lambda $ of regression loss in MBP, which is crucial to the performance of MBP. Intuitively, too small $\lambda $ may hurt the ability to predict binding affinity, while too large $\lambda $ may aggravate the label noise problem. We vary the weight coefficients $\lambda $ from $\{0, 0.01, 0.1, 0.3, 1.0\}$ and then depict the tendency curves of the test RMSE w.r.t. $\lambda $ in Figure 7. As expected, too large or too small a weight coefficient leads to worse performance in different multi-task settings (i.e. IC50 tasks, K tasks and IC50+K tasks).

### Interaction visualization and interpretation

**Figure 8:**
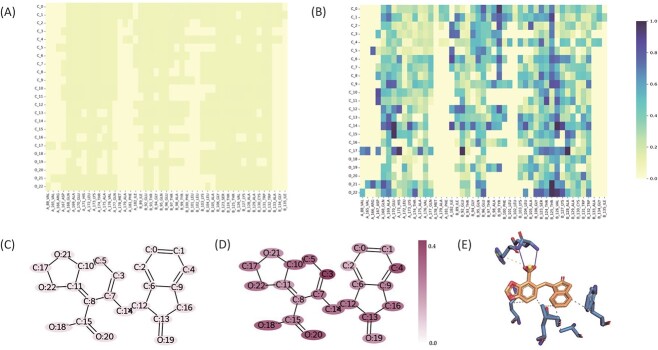
Interaction weight visualization and analysis for data 3zt2. (**A**) Protein–ligand residue–atom interaction weight before training. (**B**) Protein–ligand residue–atom interaction weight after training. (**C**) Ligand atom weight before training. (**D**) Ligand atom weight after training. (**E**) Protein–ligand detailed interaction visualized by Protein–Ligand Interaction Profiler. The gray dashed lines indicate the hydrophobic interactions and the blue solid lines indicate the hydrogen bonds.

To gain deeper insights into the contributions of ligand–protein atom–residue pairs to the final prediction, we present the visualization of atom–residue interaction weights in Figure 8. The interaction weight is defined as the absolute value of the attention weight in the Read-Out module. Furthermore, we visualize the ligand atom weights by averaging the atom–residue interaction weights that involve each specific ligand atom. As depicted in Figure 8, the interaction weights appear similar before training; however, MBP demonstrates the ability to learn some patterns after training. For instance, as shown in Figure 8D, we observe that atoms C3, C4, C5, O18 and O20 exhibit relatively stronger weights compared with other atoms. To further analyze the protein–ligand interaction, we leveraged the Protein–Ligand Interaction Profiler [101] and identified hydrophobic interactions between the C3, C4 and C5 atoms and protein residues, as well as hydrogen bonding between the O18 and O20 atoms and protein residues. These identified interactions likely contribute to the higher weights assigned to these atoms, providing partial support for our findings from the interaction weight analysis. Overall, these visualization results enhance our understanding and interpretation of the model.

## DISCUSSION AND CONCLUSION

In this study, we focused on the challenge of scarcity of high-quality training data in the PLBA prediction task. We identified three key issues: the label noise problem, the label variety problem and the missing conformation problem, which has hindered researchers from utilizing extensive datasets such as ChEMBL. To counter these challenges, we introduced the ChEMBL-Dock dataset, a collection of 313 224 3D protein–ligand complexes with experimental PLBA labels, complemented by $\sim $2.8M docked structures. Utilizing this dataset, we developed the MBP method, which mitigates the label variety and noise issues through multi-task learning and assay-specific ranking tasks. Comprehensive experiments on the PDBbind v2016 and CSAR-HiQ datasets demonstrated that our method surpassed all other baseline methods, thereby validating the effectiveness of our pre-training framework.

It is noteworthy that the current MBP model possesses significant extensibility. For instance, in this study, we utilized several basic GNNs in the shared bottom encoder of MBP to demonstrate our framework’s effectiveness. Moving forward, we are keen on implementing more advanced GNNs such as SIGN and GIGN, which could potentially enhance the performance of MBP. Additionally, we leveraged the top-1 poses for MBP pre-training. In general, we believed that more docking poses could serve as a valuable data source (just like data augmentation). The effective use of these poses may require the application of sophisticated techniques, such as negative mining in contrastive learning. We consider this a worthwhile direction to explore and plan to delve deeper into this research area. In conclusion, we hope that this work can inspire the development of other PLBA pre-training and data-cleaning frameworks.

**Key Points**

- We propose the first PLBA pre-training framework MBP, which can effectively utilize large-scale noisy data and significantly improve the accuracy and generalizability of PLBA prediction models.
- We construct a high-quality pre-training dataset, ChEMBL-Dock, based on ChEMBL, which significantly enlarges existing PLBA datasets in terms of chemical diversities.
- We show that even vanilla GNNs can significantly outperform the previous SOTA method by following the pre-training protocol in MBP.
- MBP web-server is now available for free at: https://huggingface.co/spaces/jiaxianustc/mbp. And we provide all codes and data on the online platform https://anonymous.4open.science/r/MBP-03ED.

## Data Availability

MBP web-server is now available for free at: https://huggingface.co/spaces/jiaxianustc/mbp. All codes and data are available on the online platform https://github.com/jiaxianyan/MBP.

## Author Biographies

**Jiaxian Yan** is a master’s student at the University of Science and Technology of China. His research interests include data mining and AI for Science.

**Zhaofeng Ye** got his PhD from Tsinghua University, School of Medicine. His research interests are focused on developing computational methods for drug discovery and molecular modeling. He is now a researcher at Tencent Quantum Lab.

**Zi-Yi Yang** is currently a researcher at Tencent Quantum Laboratory since 2020. She received her PhD degree in Computer Science from the Macau University of Science and Technology, Macau, China in 2020. From 2015 to 2017, she was a bioinformatics analysis engineer at BGI and China National GeneBanck, Shenzhen, China. Her research interests include artificial intelligence-aided drug discovery, bioinformatics and machine learning.

**Chengqiang Lu** is a master’s student at the University of Science and Technology of China. His research interests focus on AI for science and pre-trained language models.

**Shengyu Zhang** is a Distinguished Scientist at Tencent and the Director of Tencent Quantum Lab. He earned his PhD from Princeton University and has worked at the California Institute of Technology and The Chinese University of Hong Kong. His research interests encompass quantum computing, algorithm design, computational complexity and AI for sciences.

**Qi Liu** received his PhD from the University of Science and Technology of China (USTC). He is currently a professor in the School of Computer Science and Technology at USTC. His research interests focus on data mining and knowledge discovery. He is an Associate Editor of *IEEE Transactions on Big Data* (TBD) and *Neurocomputing*, and he was also the recipient of the National Science Fund for Excellent Young Scholars in 2019.

**Jiezhong Qiu** is a Zhejiang University - Zhejiang Lab Hundred Talents Program Researcher. He got his PhD degree from Tsinghua University in 2022. His research interests include algorithm design for large-scale information networks and representation learning for graph-structured data. He was supported by NSFC 62306290 and a funding in Zhejiang Lab (2022PE0AC04).

## A Appendix 1. Pre-training and fine-tuning procedures

In this section, to facilitate other researchers to reproduce our results, we provide the pseudocode of MBP’s pre-training procedure and fine-tuneing procedure in Algorithms 1 and 2.

## B Appendix 2. Construction of Multi-Graph

Protein graphs and ligand graphs only contain intramolecular connections, and intermolecular connections are available in protein–ligand interaction graphs. We present the pseudocode of the construction process of multi-graph in Algorithm 3.

## C Appendix 3. Experiment Details

### C.1 Hardwares

We pre-trained our model on one NVIDIA A100-PCIE-40GB for about 16 h and fine-tuned for about 1 h.

### C.2 Parameter settings

When constructing multi-graph input, we set the intramolecular cutoff distance for ligand $cut^L$ to 5.0${{\AA }}$ and intramolecular cutoff distance for protein $cut^P$ to 8.0${{\AA }}$, with an intermolecular cutoff distance $cut^I$=12.0${{\AA }}$. For determining the hyperparameters of MBP, grid search was performed in Table C1.

**Table C1:** The hyperparameter options we searched through for MBP. The final parameters are marked in bold

Parameter	Search Sapce
Ranking task weight	0.0, 0.01, 0.1, **0.3**, 1.0
Hidden dimension	64, **128**, 256
Number of layers	2, **3**, 4
Batch Size	32, 64, **128**, 256
Dropout	**0.1**
Learning rate	**0.001**

### C.3 Evaluation metrics

Here, we give the formal formulas of the evaluation metrics mentioned in the experiments section

(C.1)
\begin{align*} & RMSE=\sqrt{\frac{1}{|D|}\sum_{i=1}^{|D|}(\hat{y_i}-y_i)^2}, \end{align*}

(C.2)
\begin{align*} & MAE=\frac{1}{|D|}\sum_{i=1}^{|D|}|\hat{y_i}-y_i|, \end{align*}

(C.3)
\begin{align*} & SD=\sqrt{\frac{1}{|D|-1}\sum_{i=1}^{|D|}[y_i-(a+b\hat{y_i})]^2}, \end{align*}

(C.4)
\begin{align*} & R=\frac{ \sum_{i=1}^{|D|} (\hat{y_i}-\bar{\hat{y_i}}) (y_i-\bar{y_i})} {\sqrt{\sum_{i=1}^{|D|} (\hat{y_i}-\bar{\hat{y_i}})^2 (y_i-\bar{y_i})^2}},\end{align*}

where $a$ and $b$ are the intercepts and the slope of the regression line, respectively.

## D Appendix 4. Quality of ChEMBL-Dock

Directly evaluating the quality of ChEMBL-Dock is difficult as no co-crystal complex structures are available for comparison. Since the ChEMBL-Dock dataset was constructed entirely using the docking software SMINA, a quantitative evaluation of the ChEMBL-Dock dataset can be considered somewhat equivalent to a quantitative evaluation of the docking software SMINA, which is more feasible. To evaluate SMINA’s capability, we followed recent work on molecular docking [54, 55, 84] by performing site-specific docking on the time-split test set of PDBbind v2020, consisting of 363 data points, using the same settings as employed for constructing the ChEMBL-Dock dataset. To evaluate the performance of SMINA, we calculated the Root Mean Square Deviation metric and centroid distance metric between the top-1 docked structures and the corresponding PDBbind co-crystal structures. The results of this evaluation are presented in Table C2. We observed that the mean RMSD between top-1 docked structures and co-crystal structures is only 5.2 Å, and for 31.0% of the data points, the RMSD is less than 2.0 Å. For 88.5% data points, their centroid distance is less than 5.0 Å. These results provide evidence that the computationally generated structures possess reasonably good quality, rendering them suitable for use.

**Table C2:** Performance of SMINA on the PDBbind v2020 time-split test set

Method	RMSD			Centroid Distance		
	% below 2 Å	% below 5 Å	Mean	% below 2 Å	% below 5 Å	Mean
SMINA	31.0	49.2	5.2	59.2	88.5	2.2

## References

[1] Rizzuti B, Grande F. Chapter 14- virtual screening in drug discovery: a precious tool for a still-demanding challenge. In: Pey AL (ed). Protein Homeostasis Diseases. Academic Press, United States, 2020, 309–27.

[2] Seo S, Choi J, Park S, Ahn J. Binding affinity prediction for protein-ligand complex using deep attention mechanism based on intermolecular interactions. BMC Bioinformatics 2021;22:542. 34749664 10.1186/s12859-021-04466-0 PMC8576937

[3] Jacob L, Vert J-P. Protein-ligand interaction prediction: an improved chemogenomics approach. Bioinformatics 2008;24:2149–56. 18676415 10.1093/bioinformatics/btn409 PMC2553441

[4] Deng Y, Roux B. Computations of standard binding free energies with molecular dynamics simulations. J Phys Chem B 2009;113(8):2234–46. 19146384 10.1021/jp807701h PMC3837708

[5] Jumper JM, Evans R, Pritzel A, et al. Highly accurate protein structure prediction with alphafold. Nature 2021;596:583–9. 34265844 10.1038/s41586-021-03819-2 PMC8371605

[6] Yuan Q, Chen K, Yimin Y, et al. Prediction of anticancer peptides based on an ensemble model of deep learning and machine learning using ordinal positional encoding. Brief Bioinform 2023;24:bbac630. 10.1093/bib/bbac630 36642410

[7] Tran TO, Vo TH, Le NQK. Omics-based deep learning approaches for lung cancer decision-making and therapeutics development. Brief Funct Genomics 2023:elad031. 10.1093/bfgp/elad031 37519050

[8] Stepniewska-Dziubinska MM, Zielenkiewicz P, Siedlecki P. Development and evaluation of a deep learning model for protein-ligand binding affinity prediction. Bioinformatics 2018;34:3666–74. 29757353 10.1093/bioinformatics/bty374 PMC6198856

[9] Zheng L, Fan J, Yuguang M. Onionnet: a multiple-layer intermolecular-contact-based convolutional neural network for protein-ligand binding affinity prediction. ACS Omega 2019;4:15956–65. 31592466 10.1021/acsomega.9b01997 PMC6776976

[10] Chen L, Tan X, Wang D, et al. Transformercpi: improving compound-protein interaction prediction by sequence-based deep learning with self-attention mechanism and label reversal experiments. Bioinformatics 2020;36:4406–14. 32428219 10.1093/bioinformatics/btaa524

[11] Jiang D, Hsieh C-Y, Zhenxing W, et al. Interactiongraphnet: a novel and efficient deep graph representation learning framework for accurate protein–ligand interaction predictions. J Med Chem 2021;64:18209–32. 34878785 10.1021/acs.jmedchem.1c01830

[12] Li S, Zhou J, Tong X, et al. Structure-aware interactive graph neural networks for the prediction of protein-ligand binding affinity. KDD 2021;21.

[13] Jiménez J, Skalic M, Martinez-Rosell G, De Fabritiis G. Kdeep: protein–ligand absolute binding affinity prediction via 3d-convolutional neural networks. J Chem Inf Model 2018;58(2):287–96. 29309725 10.1021/acs.jcim.7b00650

[14] Hassan-Harrirou H, Zhang C, Lemmin T. Rosenet: improving binding affinity prediction by leveraging molecular mechanics energies with an ensemble of 3d convolutional neural networks. J Chem Inf Model 2020;60(6):2791–802. 32392050 10.1021/acs.jcim.0c00075

[15] Jones D, Kim H, Zhang X, et al. Improved protein-ligand binding affinity prediction with structure-based deep fusion inference. J Chem Inf Model 2020;61:1583–92. 10.1021/acs.jcim.0c01306 33754707

[16] Liu Z, Minyi S, Han L, et al. Forging the basis for developing protein-ligand interaction scoring functions. Acc Chem Res 2017;50(2):302–9. 28182403 10.1021/acs.accounts.6b00491

[17] Zhang Z, Liu Q, Wang H, et al. Motif-based graph self-supervised learning for molecular property prediction. In: NeurIPS ‘21, 2021, vol 34, pp 15870–15882, .

[18] Maziarka L, Danel T, Mucha S, et al. Molecule attention transformer arXiv preprint arXiv:2002.08264. 2020. 10.1186/s13321-023-00789-7 PMC10765783 38173009

[19] Rong Y, Bian Y, Xu T, et al. Self-supervised graph transformer on large-scale molecular data. In NeurIPS ‘20, 2020, pp 12559–12571.

[20] Zhu J, Xia Y, Lijun W, et al. Unified 2d and 3d pre-training of molecular representations. KDD 2022;22.

[21] Fang X, Liu L, Lei J, et al. Chemrl-gem: geometry enhanced molecular representation learning for property prediction. Nat Mach Intell 2021;4:127–34.

[22] Unsal S, Atas H, Albayrak M, et al. Learning functional properties of proteins with language models. Nat Mach Intell 2022;4:227–45.

[23] Roshan M Rao, J L, Verkuil R, et al. Msa transformer. In ICML ‘21, pp 8844–8856.

[24] Elnaggar A, Heinzinger M, Dallago C, et al. Prottrans: towards cracking the language of lifes code through self-supervised deep learning and high performance computing. TPAMI ‘21 2021;44(10):7112–27. 10.1109/TPAMI.2021.3095381 34232869

[25] Gaulton A, Bellis LJ, Patrícia Bento A, et al. Chembl: a large-scale bioactivity database for drug discovery. Nucleic Acids Res 2011;40:D1100–7. 21948594 10.1093/nar/gkr777 PMC3245175

[26] Liu T, Lin Y, Wen X, et al. Bindingdb: a web-accessible database of experimentally determined protein-ligand binding affinities. Nucleic Acids Res 2006;35:D198–201. 17145705 10.1093/nar/gkl999 PMC1751547

[27] Luo H, Xiang Y, Fang X, et al. Batchdta: implicit batch alignment enhances deep learning-based drug–target affinity estimation. Brief Bioinform 2022;23(4). 10.1093/bib/bbac260 35794723

[28] Papadatos G, Gaulton A, Hersey A, Overington JP. Activity, assay and target data curation and quality in the chembl database. J Comput Aided Mol Des 2015;29:885–96. 26201396 10.1007/s10822-015-9860-5 PMC4607714

[29] Crawshaw M . Multi-task learning with deep neural networks: a survey arXiv preprint arXiv:2009.09796. 2020.

[30] Wang M, Guo Y, Zhao Z, et al. Torr. Mp2: A momentum contrast approach for recommendation with pointwise and pairwise learning. In SIGIR ‘22, 2022.

[31] Cinar YG, Renders J-M. Adaptive pointwise-pairwise learning-to-rank for content-based personalized recommendation. RecSys 2020;20.

[32] Lei Y, Li W, Ziyu L, Zhao M. Alternating pointwise-pairwise learning for personalized item ranking. CIKM 2017;17.

[33] Kipf T N and Welling M. Semi-supervised classification with graph convolutional networks. In ICLR ‘17, 2017.

[34] Xu K, Hu W, Leskovec J, Jegelka S. *How powerful are graph neural networks?* *In* ICLR ‘19, 2019.

[35] Veličković P, Cucurull G, Casanova A, Romero A, et al. Graph attention networks. In ICLR ‘18, 2018.

[36] Han J, Rong Y, Xu T, Huang W. Geometrically equivariant graph neural networks: a survey arXiv preprint arXiv:2202.07230. 2022.

[37] Xiong Z, Wang D, Liu X, et al. Pushing the boundaries of molecular representation for drug discovery with graph attention mechanism. J Med Chem 2020;63:8749–60. 31408336 10.1021/acs.jmedchem.9b00959

[38] Gohlke H, Hendlich M, Klebe G. Knowledge-based scoring function to predict protein-ligand interactions. J Mol Biol 2000;295(2):337–56. 10623530 10.1006/jmbi.1999.3371

[39] Trott O, Olson AJ. Autodock vina: improving the speed and accuracy of docking with a new scoring function, efficient optimization, and multithreading. J Comput Chem 2010;31(2):455–61. 19499576 10.1002/jcc.21334 PMC3041641

[40] Wang R, Lai L, Wang S. Further development and validation of empirical scoring functions for structure-based binding affinity prediction. J Comput Aided Mol Des 2002;16:11–26. 12197663 10.1023/a:1016357811882

[41] Grinter SZ, Zou X. Challenges, applications, and recent advances of protein-ligand docking in structure-based drug design. Molecules 2014;19:10150–76. 25019558 10.3390/molecules190710150 PMC6270832

[42] Pason LP, Sotriffer CA. Empirical scoring functions for affinity prediction of protein-ligand complexes. Molecular Informatics 2016;35:541–8. 27870243 10.1002/minf.201600048

[43] Eldridge MD, Murray CW, Auton TR, et al. Empirical scoring functions: I. The development of a fast empirical scoring function to estimate the binding affinity of ligands in receptor complexes. J Comput Aided Mol Des 1997;11:425–45. 9385547 10.1023/a:1007996124545

[45] Temiz NA, Trapp AC, Prokopyev OA, Camacho CJ. Optimization of minimum set of protein-dna interactions: a quasi exact solution with minimum over-fitting. Bioinformatics 2009;26:319–25. 19965883 10.1093/bioinformatics/btp664 PMC2815656

[46] Ballester PJ, Mitchell JBO. A machine learning approach to predicting protein–ligand binding affinity with applications to molecular docking. Bioinformatics 2010;26(9):1169–75. 20236947 10.1093/bioinformatics/btq112 PMC3524828

[47] Kinnings SL, Liu N, Tonge PJ, et al. A machine learning-based method to improve docking scoring functions and its application to drug repurposing. J Chem Inf Model 2011;51(2):408–19. 21291174 10.1021/ci100369f PMC3076728

[48] Pellicani F, Dal Ben D, Perali A, Pilati S. Machine learning scoring functions for drug discovery from experimental and computer-generated protein-ligand structures: towards per-target scoring functions. Molecules 2022;28. 10.3390/molecules28041661 PMC9966217 36838647

[50] Nguyen T, Le H, Quinn TP, et al. Graphdta: predicting drug–target binding affinity with graph neural networks. Bioinformatics 2021;37(8):1140–7. 33119053 10.1093/bioinformatics/btaa921

[51] Danel T, Spurek P, Tabor J, et al. Spatial graph convolutional networks. In ICONIP ‘20, 2020, pp 668–675.

[52] Gasteiger J, Groß J, Günnemann S. Directional message passing for molecular graphs. In *I* CLR ‘20, 2020.

[53] Song Y, Zheng S, Niu Z, et al. Communicative representation learning on attributed molecular graphs. In IJCAI ‘20, 2020.

[54] Kramer C, Gedeck P. Leave-cluster-out cross-validation is appropriate for scoring functions derived from diverse protein data sets. J Chem Inf Model 2010;50(11):1961–9. 20936880 10.1021/ci100264e

[55] Dunbar JB, Smith RD, Damm-Ganamet KL, et al. Csar data set release 2012: ligands, affinities, complexes, and docking decoys. J Chem Inf Model 2013;53:1842–52. 23617227 10.1021/ci4000486 PMC3753885

[56] Lu W, Wu Q, Zhang J, et al. TANKBind: Trigonometry-aware neural networks for drug-protein binding structure prediction. In NeurIPS ‘22, 2022.

[57] Stärk H, Ganea O-E, Pattanaik L, et al. Equibind: Geometric deep learning for drug binding structure prediction. In ICML ‘22, 2022.

[58] Berman HM, Westbrook JD, Feng Z, et al. The protein data bank. Acta Crystallogr D Biol Crystallogr 2000;58 Pt **58**(1):899–907. 10.1107/s0907444902003451 12037327

[59] Meng X, Zhang H-X, Mezei M, Cui M. Molecular docking: a powerful approach for structure-based drug discovery. Curr Comput Aided Drug Des 2011;7(2):146–57. 21534921 10.2174/157340911795677602 PMC3151162

[60] Francoeur PG, Masuda T, Sunseri J, et al. Three-dimensional convolutional neural networks and a cross-docked data set for structure-based drug design. J Chem Inf Model 2020;60:4200–15. 32865404 10.1021/acs.jcim.0c00411 PMC8902699

[61] Peng X, Luo S, Guan J, et al. Pocket2mol: Efficient molecular sampling based on 3d protein pockets. In ICML ‘22, 2022.

[62] Luo S, Chen T, Xu Y, et al. One transformer can understand both 2d & 3d molecular data. In ICLR ‘23, 2023.

[63] Lee J, Yoon W, Kim S, et al. Biobert: a pre-trained biomedical language representation model for biomedical text mining. Bioinformatics 2019;36:1234–40. 10.1093/bioinformatics/btz682 PMC7703786 31501885

[64] Lin Z, Akin H, Rao R, et al. Language models of protein sequences at the scale of evolution enable accurate structure prediction bioRxiv. 2022.

[65] Zhou G, Gao Z, Ding Q, et al. Uni-mol: A universal 3d molecular representation learning framework. In ICLR ‘23, 2023.

[66] Cao Z, Qin T, Liu T-Y, et al. Learning to rank: from pairwise approach to listwise approach. In ICML ‘07, 2007.

[67] Köppel M, Segner A, Wagener M, et al. Pairwise learning to rank by neural networks revisited: Reconstruction, theoretical analysis and practical performance. In ECML/PKDD ‘19, 2019, pp 237–252.

[68] Liu T-Y . Learning to rank for information retrieval. *Foundations and trends* ®. Information Retrieval 2009;3(3):225–331.

[69] Rendle S, Freudenthaler C, Gantner Z, Schmidt-Thieme L. Bpr: Bayesian personalized ranking from implicit feedback. In UAI ‘09, 2009, pp 452–461.

[70] Burges C, Shaked T, Renshaw E, Lazier A, et al. Learning to rank using gradient descent. In ICML ‘05, 2005, pp 89–96.

[71] Zheng Z, Chen K, Sun G, Zha H. A regression framework for learning ranking functions using relative relevance judgments. In SIGIR ‘07, 2007, pp 287–294.

[72] Lee C-P, Lin C-J. Large-scale linear ranksvm. Neural Comput 2014;26(4):781–817. 24479776 10.1162/NECO_a_00571

[73] Bliss CI . Some principles of bioassay. Am Sci 1957;45(5):449–66.

[74] Landrum G, Tosco P, Kelley B, et al. rdkit/rdkit: 2022_03_4 (q1 2022) release. In: , 2022.

[75] Koes DR, Baumgartner MP, Camacho CJ. Lessons learned in empirical scoring with smina from the csar 2011 benchmarking exercise. J Chem Inf Model 2013;53(8):1893–904. 23379370 10.1021/ci300604z PMC3726561

[76] Meli R, Morris GM, Biggin P. Scoring functions for protein-ligand binding affinity prediction using structure-based deep learning: a review. Frontiers in bioinformatics 2022;2:57. 10.3389/fbinf.2022.885983 PMC7613667 36187180

[77] Natarajan N, Dhillon IS, Ravikumar PK, Tewari A. Learning with noisy labels. NeurIPS 2013;13:26.

[78] Caruana R . Multitask learning: a knowledge-based source of inductive bias1. ICML ‘93 1993;41–8.

[79] Kwon Y, Shin W-H, Ko J, Lee J. Ak-score: accurate protein-ligand binding affinity prediction using an ensemble of 3d-convolutional neural networks. Int J Mol Sci 2020;21. 10.3390/ijms21228424 PMC7697539 33182567

[80] Moon S, Zhung W, Yang S, et al. Pignet: a physics-informed deep learning model toward generalized drug-target interaction predictions. Chem Sci 2020;13:3661–73. 10.1039/d1sc06946b PMC8966633 35432900

[81] Yan X, Liu Y. Graph-sequence attention and transformer for predicting drug-target affinity. RSC Adv 2022;12:29525–34. 36320763 10.1039/d2ra05566j PMC9562047

[82] Wang H, Guo F, Mengyan D, et al. A novel method for drug-target interaction prediction based on graph transformers model. BMC Bioinformatics 2022;23:459. 36329406 10.1186/s12859-022-04812-w PMC9635108

[83] O’Boyle NM, Banck MS, James CA, et al. Open babel: an open chemical toolbox. J Chem 2011;3:33–3. 10.1186/1758-2946-3-33 PMC3198950 21982300

[84] Ganea O-E, Huang X, Bunne C, Bian Y, et al. Independent se(3)-equivariant models for end-to-end rigid protein docking. In ICLR ‘22, 2022.

[85] Klicpera J, Groß J, Günnemann S. Directional message passing for molecular graphs. In ICLR ‘20, 2020.

[86] Corso G, Stärk H, Jing B, Barzilay R, et al. Diffdock: Diffusion steps, twists, and turns for molecular docking. In ICLR ‘23, 2023.

[87] Zhang H, Huang Y, Bei Z, et al. Inter-residue distance prediction from duet deep learning models. Front Genet 2022;13. 10.3389/fgene.2022.887491 PMC9148999 35651930

[88] Sheng, Wang S, Sun ZL, Zhang R, Jinbo X. Accurate de novo prediction of protein contact map by ultra-deep learning model. PLoS Comput Biol 2016;13. 10.1371/journal.pcbi.1005324 PMC5249242 28056090

[89] Muegge I, Martin YC. A general and fast scoring function for protein-ligand interactions: a simplified potential approach. J Med Chem 1999;42(5):791–804. 10072678 10.1021/jm980536j

[90] Gilmer J, Schoenholz SS, Riley PF, et al. Neural message passing for quantum chemistry. In ICML ‘17, 2017, pp 1263–1272.

[91] William L. Hamilton, ZY, and Leskovec J. Inductive representation learning on large graphs. In NeurIPS ‘17, 2017.

[92] Xu K, Li C, Tian Y, Sonobe T, et al. Representation learning on graphs with jumping knowledge networks. In ICML ‘18, 2018, pp 5453–5462.

[94] Lim J, Ryu S, Park K, et al. Predicting drug–target interaction using a novel graph neural network with 3d structure-embedded graph representation. J Chem Inf Model 2019;59(9):3981–8. 31443612 10.1021/acs.jcim.9b00387

[95] Moesser MA, Klein D, Boyles F, et al. Protein-ligand interaction graphs: learning from ligand-shaped 3d interaction graphs to improve binding affinity prediction bioRxiv. 2022;2022–03.

[96] Zhang S, Liu Y, Xie L. Efficient and accurate physics-aware multiplex graph neural networks for 3d small molecules and macromolecule complexes arXiv preprint arXiv:2206.02789. 2022.

[97] Zhang Y, Zhou G, Wei Z, Hongteng Xu. Predicting protein-ligand binding affinity via joint global-local interaction modeling. 2022 IEEE International Conference on Data Mining (ICDM), 2022, pp 1323–1328.

[98] Ruiqiang L, Wang J, Li P, et al. Improving drug-target affinity prediction via feature fusion and knowledge distillation. Brief Bioinform 2023;24. 10.1093/bib/bbad145 37099690

[99] Wang K, Zhou R, Tang J, Li M. Graphscoredta: optimized graph neural network for protein-ligand binding affinity prediction. Bioinformatics 2023;39. 10.1093/bioinformatics/btad340 PMC10243863 37225408

[100] Zhang X-j, Gao H, Wang H, et al. Planet: a multi-objective graph neural network model for protein-ligand binding affinity prediction bioRxiv. 2023. 10.1021/acs.jcim.3c00253 37319418

[101] Yang Z, Zhong W, Lv Q, et al. Geometric interaction graph neural network for predicting protein–ligand binding affinities from 3d structures (gign). The Journal of Physical Chemistry Letters 2023;14(8):2020–33. 36794930 10.1021/acs.jpclett.2c03906

[102] Diederik P. Kingma and Jimmy Ba. Adam: A method for stochastic optimization. In ICLR ‘15, 2015.

[103] Feinberg EN, Joshi EM, Pande VS, Cheng AC. Improvement in admet prediction with multitask deep featurization. J Med Chem 2020;63:8835–48. 32286824 10.1021/acs.jmedchem.9b02187

[104] Salentin S, Schreiber S, Haupt VJ, et al. Plip: fully automated protein-ligand interaction profiler. Nucleic Acids Res 2015;43:W443–7. 25873628 10.1093/nar/gkv315 PMC4489249

